# The role of telephone consultations in psychiatry

**DOI:** 10.1192/bjo.2021.889

**Published:** 2021-06-18

**Authors:** Jonathan Packer, Emma Fisher, Anne-Marie Simons, Kirsty Bolochowecki, Benita Roff, Sanjay Khurmi, Luke Jones

**Affiliations:** 1 Herefordshire and Worcestershire Health and Care Trust; 2 Coventry and Warwickshire Partnership Trust; 3 Warwick Medical School

## Abstract

**Aims:**

Telephone consultations have been in clinical use since the early 1960s and are increasing in frequency and importance in many areas of medicine. With the advent of the COVID-19 pandemic in 2020, the use of telemedicine consultations increased dramatically alongside utilization of other digital technologies. Despite promise and potential advantages for clinicians (including remote working, improved time management and safety) there are known drawbacks to telephone consultations for psychiatrists. This includes limitations to assessments of mental state and risk, with loss of non-verbal communication often cited as a point in favour of more sophisticated technologies such as video calling. By adopting telephone consultations to a greater extent during the initial months of the COVID-19 pandemic in the Coventry Crisis Resolution and Home Treatment Team (CRHTT), we aimed to assess the patient experience in telehealth, through a patient survey.

**Method:**

After an initial assessment or follow-up consultation with a medical practitioner from the crisis team, patients were invited to take part in a short questionnaire with a member of the administration staff. This consisted of eight questions on a Likert scale and three open questions for comments. Results were collated and analyzed via Microsoft Excel.

**Result:**

Most patients found the telephone consultations satisfactory, with more than 90% returning positive scores in understanding, convenience and overall satisfaction. All patients felt listened to and that their confidentiality was maintained; with all but one respondent willing to engage in further consultations via the telephone. Negative scores were typically returned for practical telephonic problems including poor signal, interference and background noise. In their comments patients expressed largely positive views about their experience with their clinician; analysis revealed key insights into the patient experience, demonstrating the convenience, comfort and flexibility possible with ‘telepsychiatry’.

**Conclusion:**

Patient experience of telemedicine in a UK psychiatric crisis team is mostly positive, with clear advantages for both patients and clinicians. Our results show telephone consultations can be expanded to new patient assessments alongside follow-ups, enabling the team to reach a greater number of service users. This includes service users who are housebound due to infirmity, required to shield or have significant anxiety about the pandemic.

